# Development and Dissolution Study of a β-Galactosidase Containing Drinking Straw

**DOI:** 10.3390/pharmaceutics14040769

**Published:** 2022-03-31

**Authors:** Márton Király, Konrád Sántha, Barnabás Kállai-Szabó, Kriszta Mariann Pencz, Krisztina Ludányi, Nikolett Kállai-Szabó, István Antal

**Affiliations:** 1 Department of Pharmaceutics, Semmelweis University, Hőgyes E. Street 7–9, 1092 Budapest, Hungary; kiraly.marton@pharma.semmelweis-univ.hu (M.K.); santha.konrad@pharma.semmelweis-univ.hu (K.S.); 2 ViteCer Ltd., Ipartelepi Street 8/b, 2700 Cegled, Hungary; barnabas.kallai-szabo@vitecer.com (B.K.-S.); kriszta.pencz@vitecer.com (K.M.P.)

**Keywords:** β-galactosidase, drinking straw, dissolution, pellets

## Abstract

Today, in addition to many different physicochemical and pharmacological properties of the active ingredients and excipients, the developer of a pharmaceutical formulation must take into account several factors during the formulation process in order for the patient to cooperate to use the formulation accurately. One of the innovative solutions in paediatrics may be the use of medicated drinking straws. For our studies, we successfully prepared lactase-containing, rapid disintegration particles by two techniques commonly used in the pharmaceutical industry. The simulation of the usage of the filled straws was presented from a new perspective for the patient by an in vitro method. The effect of the temperature of the liquid used during the administration of the straw and the effect of the frequency during the application on the dissolution rate were investigated. According to our results, in the case of a straw containing rapidly dissolving particles, the temperature of the used liquid and the mode of administration (frequency) play a significant role in the release rate from the composition.

## 1. Introduction

Oral administration of the active ingredients is still the most commonly used route of administration [1,2]. Many oral formulations are commercially available not only in various strengths, but also in several dosage forms. One reason for this phenomenon is that the users or patients of the drug product are different from each other [3]. The difference can be due to many factors, e.g., patients may differ in terms of gender, health status, and age. Medication for children and the elderly is a special field, as significant differences compared to adults need to be considered. This is why there is a growing need today to develop age-appropriate oral drug delivery systems that take age-related requirements into account. For example, children are more likely to prefer different flavours, colours, and dosage forms compared to adults [4]. The paediatric part of the population can also be considered very heterogeneous, which also affects therapy, therefore subgroups also exist within every age group of children. The division of various agencies may indeed differ in this respect, but for example the International Conference Harmonization of Technical Requirements for the Registration of Pharmaceuticals for Human Use model is generally accepted and endorsed by the World Health Organization (WHO) [5]. The analysis of Alessandrini et al. presents the results of research in six European countries. It has also been statistically proven that liquid dosage forms are significantly popular among younger children, but this trend is decreasing with age, while capsules and tablets are coming to the fore [6]. Therefore, excipients should be selected with due care in the design of paediatric formulations, as there are several pharmacokinetic and pharmacodynamic differences with age [7,8].

For children, the elderly, and those with having difficulty swallowing-dysphagia-taking a larger tablet or capsule can be problematic, resulting in decreased patient compliance during therapy, thus increasing the need to produce formulations that are easy to swallow. Minitablets, devices for accurate dosing and for small-size particulates (smart mini-tablet dispenser; Sympfiny™) have appeared on the market, and more and more orodispersible dosage forms (tablets; granules, film/strips), sprinkle formulations have become available [9,10]. Orodisperse systems are considered a ready-to-use formula, while sprinkle formulations require manipulation [11]. According to a summary published by Lee et al. in 2020, 65 original sprinkle medications were available in the United States, and their number will increase in the following years as the current trend dictates. These sprinkle formulations can be found in several dosage forms (tablets, powders, granules, IR capsules, extended-release capsules, and multiparticulate carrier systems), mixed with soft food, or added to liquid before administration [12]. One of the innovative drug delivery solutions specifically in paediatrics is particles loaded into drinking straws.

The drinking straw as a dosage form is no stranger to children. Although the usage of disposable single-use plastic (SUP) straws has been forbidden in Hungary since the 3rd of June, as well as in the Member States of the European Union. The popularity of the sipping devices is shown by the fact that straws made of many other materials (paper, metal, glass, silicone, PLA-polylactic acid, edible pasta) were released for commercial use [13,14,15]. The pharmaceutical product, called ClaroSip^®^ was introduced and produced by Grünenthal GmbH in Germany between 2006 and 2009 and was also available in Hungary. The white or yellowish, almost spherical, coated, and flavoured clarithromycin-loaded granules were filled into a transparent PP straw. The straw had a white cap on one side and a polyester/polyolefin porous plunger on the other. The prescribed, pre-measured dose could be easily ingested by the patient with his preferred fibre-free liquid, which could be either a carbonated beverage or milk (below 3.5%) fat content [16]. Although ClaroSip is not currently available, it does not mean that the straw as a dosage form has disappeared. The XStraw^®^ as a pharmaceutical innovation for new active ingredients was introduced by DS-Technology GmbH, a subsidiary of Harro Höffliger [11,17]. Simsic et al. coated paracetamol crystals using different polymers (water-soluble and enteric) then granulated with a trehalose/erythritol mixture and finally examined the taste coverage during intake. According to their results, Kollicoat IR and Kollicoat Protect are the most suitable materials for masking the unpleasant taste of paracetamol particles during the sipping simulations [18]. In both studies, patients swallow small particles during the admission. There are also Hungarian aspects to the development/production of straws filled with particles containing active ingredients. Farkas et al. prepared medicated straws containing electrospun fibres and studied carvedilol dissolution at a constant suction rate [19]. A patented multivitamin straw formulation for children (Vitasip Kids) has been marketed various tastes in several European countries. In addition to the vitamin, a straw, containing hyaluronic acid and collagen, the building materials of skin tissue, is also available [20,21]. However, in the case of Vitasip products, meanwhile the patient swallows liquid, the great advantage over liquid dosage forms is that the dosage is simple and preservative-free.

In the past decades, the application of several enzymes became essential for different industries, in healthcare and analytics. Lipase, amylase, and protease enzymes for pancreatic dysfunction, β-galactosidases for lactose intolerance are used as substitution for their enzyme deficiency since it is the only possibility to treat these diseases. Galactosidase enzymes, like lactases that catalyse the breakup of galactosyl bonds, are being applied in a wide area in the food and pharmaceutical industry due to the increasing incidence of intolerance to dairy products caused by lactase enzyme deficiency. These dairy products play an important role in gastronomy in many parts of the world, so an irregularity during their metabolism can lead to a serious deterioration in the quality of life [22]. The treatment of this disease involves the administration of various medications containing lactase. Since this disease affects every age group and its prevalence can be more than 60% amongst the population of the world, it is important to find easy and accessible admission methods for everyone [23].

In the present study, we produced a straw through which the patient swallows the fluid. The enzyme lactase was chosen as the model active substance. The straws were filled with rapidly disintegrating, nearly spherical shaped particles. The particles were prepared by both layering and extrusion methods, and their main physical parameters were examined separately. The mode of administration was simulated with an array for testing the release of drug from the straws, and the released lactase and the indigo carmine content were determined. The kinetic parameters of the in vitro drug release profiles were performed by model-dependent (Weibull) evaluation. An experimental design was used, in order to investigate the effect of the temperature of the fluid and the suction force on the kinetic parameters of drug release. According to our results, we were able to produce particles with rapid disintegration that had appropriate physical parameters. When applied to the straw the kinetic parameters of the dissolution profiles were examined.

## 2. Materials and Methods

### 2.1. Materials

The powder of β-galactosidase enzyme (Opti-lactase A-50 powder) derived from Aspergillus oryzae was purchased from Optiferm Gmbh (Oy-Mittleberg, Germany). For the production of the lactase-containing particles in the straw, crystalline sugar (Südzucker AG, Raffinade RG, Batch: L119831800), corn starch (Hungrana, Hungramid F, Batch: H10782451), banana aroma (Taste-point d.o.o., Banana 22035 NF, Batch: 21A0987813) as a flavoring agent, and indigo carmine (E132; Sensient Food Colors Hungary Kft., Batch: 5577515) as a colouring agent were used as excipients.

O-nitrophenyl-β-D-galactopyranoside (ONPG) obtained from VWR International Kft. (Debrecen, Hungary) was used to measure lactase content. Ultrapure water obtained from the MilliRO-hMilliQ-system (Merck Millipore, Burlington, MA, USA) was used throughout all measurements.

### 2.2. Preparation of Fast Dissolving Particles

The particles were produced with two different technological methods. Extrusion-spheronization technique was used to produce particles with a homogeneous (matrix) structure. The particulates made by layering process have heterogeneous, layered structure.

#### 2.2.1. Extrusion-Spheronisation Method for the Preparation of Matrix Structured Particles

The crystalline sugar was milled under 200 μm using a turbo sugar mill (custom made, I-250, machine number: 2021/12 with 0.2 mm sieve) with 1% *w*/*w* corn starch addition. Then, 99.25 g of powder mixture were wetted with 5.4 g of water containing 0.667 g of β-galactosidase, 0.084 g of indigo carmine and 0.6 g of aroma. The wetted composition was fed into the extruder part of a Caleva Multi Lab machine (Caleva Process Solutions Ltd., Sturminster Newton, UK) working at 120 rpm and extruding through die holes of 2 mm. The extrudates were then rounded for 5 min at 30 Hz in a spheroniser (Locost GSZF-AK; Locost Kft., Tiszaalpár, Hungary), and consequently dried at 40 °C in a drying cabinet for 48 h.

#### 2.2.2. Layering Process for the Preparation of Heterogeneous Structured Particulates

The batch was made using a dragee pan (custom made, material: stainless steel, opening: 300 mm, depth: 350 mm, outer diameter: 600 mm, rotation: 20 revolutions/min). Then, 1 kg of crystalline sugar was layered by 1 kg of milled sugar with 200 mL coating liquid. The coating liquid was made using 200 g of 70% *w*/*w* sugar syrup, 11 g banana flavouring agent, 13.3 g lactase enzyme, and 1.67 g of indigo carmine. The wet particulates were dried on stainless steel trays in the drying room at 45 °C for 2 days. The remaining moisture content of the dragees was under 1.2% *w*/*w*.

Both samples were then fractioned on a Retsch AS 200 vibration sieve (Retsch Gmbh, Haan, Germany) at an amplitude of 1.5 mm for 2 min, and the 2–3 mm-sized fractions were used. The amount of active substance in one dose (2000 IU) has been determined in a way to be comparable to other enzyme containing products on the market.

### 2.3. Production of Straws

First, 5 g of prepared lactase-containing particulates were weighted into the semi-closed commercially available (width = 8 mm, length = 190 mm) polypropylene drinking straws, and the samples were sealed by a custom-made straw-closing machine.

### 2.4. Physical Characterization of Particles

Shape and size analysis, image analysis of particles of both types were carried out on photomicrographs taken by a digital camera (Coolpix 4500, Nikon, Tokyo, Japan) connected to a stereomicroscope (SMZ 1000, Nikon), with a resolution of 2.3 pixels/µm. The particulates were placed on the non-shiny black surface and were illuminated from the top using a cold white coherent fibre-optic light (230 V, 185 W, 50/60 Hz, d = 5.4 mm) of halogen light source (Intralux 5000-1 type, Volpi, Dietikon, Switzerland). The images were analysed by an image processing software (ImageJ 1.53a; Wayne Rasband, National Institute of Health, Bethesda, MD, USA), and roundness, maximum Feret diameter, and projected area were used to characterize the particles. Particle size distribution was calculated from the maximum Feret diameter values. The given values presented for each type of formula (matrix, layered) are the average and standard deviation (SD) calculated from the measurement of 200 individual particulates.

Flowability and compressibility of both types of particles were measured according to the European Pharmacopoeia 10th edition [24]. Each material was tested by Pharmatest PTG (Pharmatest Apparatebau AG, Hainburg, Germany) to measure flowability, then into a STAV 2003 Stampfvolumeter (J. Engelsmann AG., Ludwigshafen am Rhein, Germany) to measure bulk density, tapped density, and Hausner ratio (HR).

Disintegration time of the particles was measured in a Pharmatron DISI 2 tablet disintegration time tester (Pharmatron AG, Thun, Switzerland). Then, 1.0 g of each type of particles were placed into each of the six tubes of the testing apparatus, then the test was carried out according to the European Pharmacopoeia [25].

### 2.5. X-ray Crystallography

Diffraction patterns were measured on PANalytical X’Pert3 Powder diffractometer (Malvern Panalytical B.V., Almelo, The Netherlands) using Cu Kα radiation with 45 kV accelerating voltage and 40 mA anode current over the range of 2–40° 2 θ with 0.0084° step size and 100 s times per step in reflection mode, spinning the sample holder by 1 s^−1^. Incident beam optics were as follows: Programmable divergence slit with 15 mm constant irradiated length, anti-scatter slit at fixed 2°. Diffracted beam optics consisted of X’Celerator Scientific ultra-fast line detector with 0.02 soller slit and programmable anti-scatter slit with 15 mm constant observed length. Data were collected by PANalytical Data Collector Application, v5.5.0.505 (Malvern Panalytical B.V., Almelo, The Netherlands).

### 2.6. Characterization of Straws

**Figure 1:**
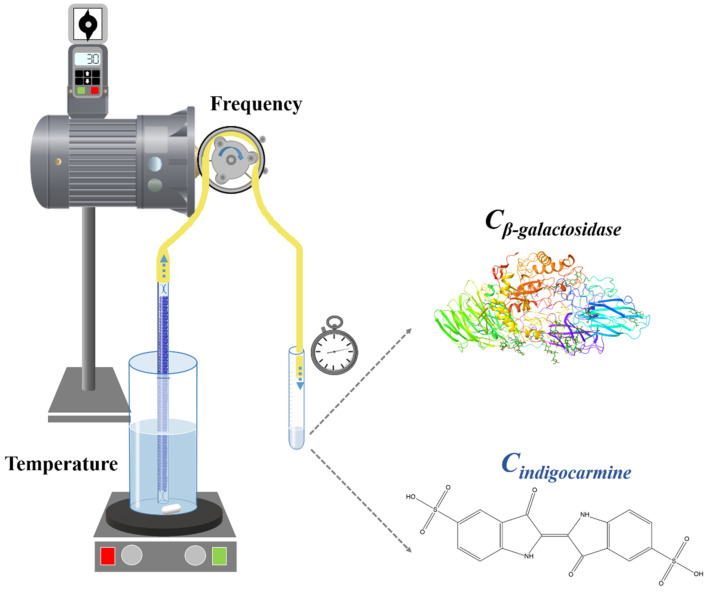
The apparatus for simulating the sipping of liquid through the straw.

The end of the straw was connected to a peristaltic pump (Locost Kft., Tiszaalpár, Hungary) developed for this purpose, which was driven by a CEMER motor (Cosgra S.A., Crespià, Spain) with 30/40 or 50 Hz rotation frequency. The flow rate generated by the different frequencies is shown in Table 1. The mass of the different liquid fractions was measured using a Sartorius BL310 balance (Sartorius Stedim Biotech, Aubagne, France) The straws filled with particles were dipped into a beaker containing 300 mL of distilled water. As the effects of the sipping rate and also the temperature of the liquid used were investigated, the measurements were performed at three various temperatures (5 °C, 20 °C, and 35 °C). The constant temperature was provided by a heated magnetic stirrer (IKA RCT basic heated magnetic stirrer equipped with IKATRON ETS-D5 contact thermometer; IKA^®^-Werke GmbH & Co., Staufen im Breisgau, Germany). The sampling was executed at the other end of the pump. The arrangement of the measurement is illustrated in Figure 1. The flow rates for the various frequencies were determined through the empty, unloaded straws: The measured value in case of 30 Hz was 72.06 ± 0.62 g/min. After increasing the frequency of the pump to 40 and 50 Hz the following values were determined 99.7 ± 1.39 and 104.76 ± 0.70 g/min, respectively (*n* = 3, mean ± SD).

The measurement lasted for 3 min, during which 7 samples were taken. The first 4 samples were taken in the first minute with 15 s intervals, then at 90 and 120 s, and finally the last at 3 min. All the measurements were performed parallel on 3 different straws.

From each sample two different dilutions (50× and 500×) were made consecutively using distilled water. The fifty-fold diluted samples were measured directly by UV spectroscopy utilizing an ATi Unicam UV2 UV/VIS Spectrometer (UNICAM, Budapest, Hungary) at 610 nm to determine the indigo carmine content. The β-galactosidase enzyme content of the samples was determined from the other dilution. The quantity of both substances was calculated applying predetermined calibration curves. The calibration standards were diluted from the stock solution to obtain six calibration levels and were ran in duplicate at the beginning of the measurement process. The linearity values were for indigo carmine R^2^ = 1.000 and for β-galactosidase R^2^ = 0.993, respectively. The lowest and highest points of the calibration curve coincided with the lower limit of quantitation (LLOQ) as well as the upper limit of quantitation (ULOQ). Intra-day accuracy and precision were assessed by evaluating five replicates of one low and one high concentrated QC samples (*n* = 5-5). Accuracy was expressed as percentage of the nominal concentration and precision was calculated as the relative standard deviation (RSD). The acceptance criteria for both parameters were set at ±15% (Table 1).

The hydrolysing activity of the examined enzyme was estimated using ONPG as substrate. ONPG is a colourless substance, which is cleaved by β-galactosidase to galactose and o-nitrophenol (ONP), the reaction is indicated by a yellow colour change. The reagent was dissolved in pH = 4.5 phosphate-citrate (McIlvaine) buffer solution. The sample and the reagent solutions were mixed in a ratio of 1:3. The samples were incubated in a water bath at 37 °C for 30 min. Aliquots were withdrawn from the reaction mixture and pipetted directly into 1 mL Na_2_CO_3_ solution to stop the enzymatic hydrolysis and to produce the maximum possible absorption due to the liberated o-nitrophenolate ion. The solution was left to cool to room temperature and the absorbance of o-nitrophenol was determined by UV-VIS spectroscopy at 420 nm. The presence of indigo carmine in such dilution at this wavelength is completely negligible.

### 2.7. Kinetic Study

The kinetic characterization of the curves obtained by simulating the sip was studied with a Weibull distribution function (Equation (1)). The Weibull distribution has long proved to be a suitable mathematical model for determining the kinetic parameters of drug release profiles with different shapes and for comparing them [26].

(1) $$M_{t}=M_{\infty }\left[1-{e^{-\left(\frac{t-t_{0}}{\tau_{d}}\right)}}^{b}\right]$$

*M_t_*-the percentage of the released lactase/indigo carmine at time; *M_∞_*-the infinite concentration of the lactase/indigo carmine in percentages; *t_0_*-dissolution lag time; *b*-curve shape parameter; *τ_d_*-time in minutes when 63.2% of the API has been dissolved.

Where *M_t_* is the % of the released lactase or indigo carmine at time *t, M_∞_* is the infinite concentration (%) of the lactase or indigo carmine, *t*_0_ is the lag-time of the dissolution, *b* is the shape parameter of the curve, and the *τ_d_* represents the time (secundum) when 63.2% of the drug has been dissolved. A *3*^2^ type face-centred factorial design was used to investigate the effects of sipping rate (frequency) as well as the temperature of liquid on the dissolution kinetics. The two independent factors as well as their three levels are shown in Table 2. The effect of the independent variables (*x*_1_ and *x*_2_) on response *y* were modelled by the following polynomial Equation (2):

(2) $$y_{}=b_{0}+b_{1}x_{1}+b_{2}x_{2}+b_{11}x_{1}^{2}+b_{22}x_{2}^{2}+b_{12}x_{1}x_{2}$$

*y*-the response; *b*_0_; *b*_1_; *b*_11_; *b*_2_; *b*_22_; *b*_12_-coefficients; *x*_1_; *x*_2_-factors.

**Table 2:** The values and codes of the different dissolution test conditions.

Coded Value	Actual Value *x*_1_ (Freuquency; Hz)	Actual Value *x*_2_ (Temperature of Liquid; °C)
−1	30	5
0	40	20
+1	50	35

Where *x* are the factors (*x*_1_: frequency of sipping during simulation; *x*_2_: temperature of liquid) and *b* parameters mark the coefficients characterizing the main (*b*_1_, *b*_2_), the quadratic (*b*_11_, *b*_22_), and the interaction effects (*b*_12_). The main effects describe the average result of the varying of one factor at a time. The interaction terms determine how the response (*y: τ_d_*) changes when two independent factors are changed simultaneously. The quadratic polynomial terms are included to investigate nonlinearity. Statistical analysis was performed using TableCurveR 3Dv4.0 (Systat Software Inc., London, UK).

## 3. Results and Discussion

### 3.1. Physical Characterization of Particles

During the formulation, the aim was to use as few excipients as possible, while providing a pleasant taste if possible, taking into account a probable paediatric use. It is important for the formula to dissolve well, leaving no remaining excipient (e.g., inert pellet core) in the straw, which makes it easier to observe and control that the full amount of the dose has been administrated. For this reason, our choice to design a sugar-based formula was the most ideal. Sugar spheres and sucrose have long been used in both the pharmaceutical and food industries. The use of sucrose in paediatric medicinal products is widely accepted [8].

Different formulas were tested during the development of the matrix structures. The popular microcrystalline cellulose for the extrusion/spheronization processes was also tested in various amounts to produce the optimal matrix structure. The particles produced could have been characterized by better shape parameters, but their disintegration time greatly increased. In order to improve the disintegration, super disintegrant was used in addition to the amount of MCC, but it did not suffice. Then, different amounts of starch were added, but this was also not appropriate because starch is practically insoluble in cold water, thus it was unsuitable for developing pellets for straws. After these steps, sugar remained as a major component in the composition. During the production, the amount of water added had to be determined, with which a sufficiently moist but still extrudable mass could be formed. Paying attention to using as little water as possible, as water removal is a critical step in the production of sugar-containing formulas, and residual water can cause the degradation of active ingredients or can lead to microbiological problems. After finding the right materials, the various extrusion/spheronization parameters were set. The matrix pellets were prepared in pilot laboratory scale (batch size: 100 g). Three batches were made from the final composition, which were then examined and mixed before being filled into the straws.

Formulations of adequate quality were obtained as the result of both production methods. Upon completion of manufacture, the activity of the finished dosage forms was compared to the starting material. In all cases, the end products retained more than 98% (Matrix: 98.22 ± 1.71%, Layered: 98.59 ± 0.86%, *n* = 10-10), of their original activity, confirming that the appropriate production conditions were chosen according to our preliminary measurements [27].

**Figure 2:**
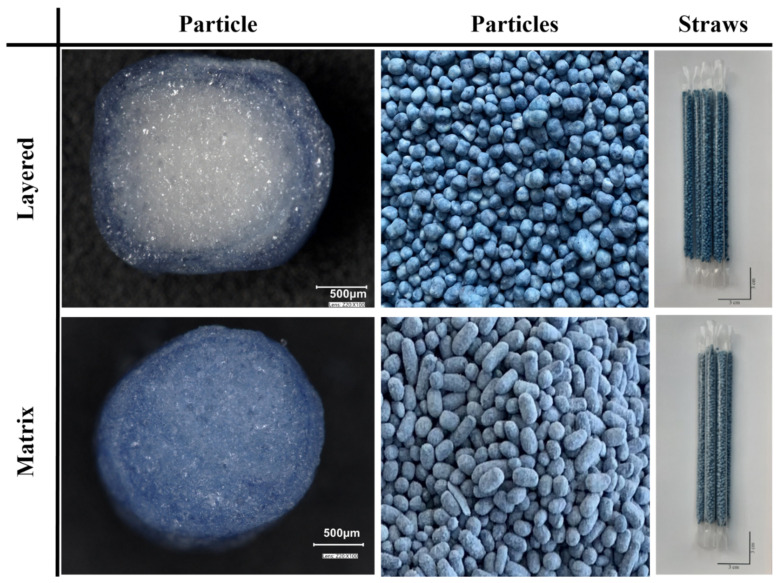
Microscopic images of the halved particles prepared by layering technique and extrusion/spheronization method. Photograph of the particles in bulk and of the straws filled with the various particles produced.

The physical properties of the particulates produced by the two methods are shown in Table 3. The results of the image analysis are included in the first three columns. The roundness of a geometric form with a perfectly spherical shape is 1. The more the value differs from 1, the more the shape of the examined object differs from the perfect sphere [28]. The figure (Figure 2) shows a cross-sectional view of the particles produced by the two methods. The difference is well observed in the figure. In the case of the layered particle, the sugar bead used as the starting core can be observed, which has a diameter of 1.00–1.60 mm according to the manufacturer’s specification. The matrix structure can be recognized in Figure 2, where there is no such heterogeneous division in the structure.

The pellets were successfully loaded into straws. Photographs of the filled straws are shown in Figure 2. As the HR value of the produced particles is below 1.11 [24], the matrix and layered particles both had excellent flow properties, predicting the fast filling process of the empty straws.

Comparing the particulates produced by the two methods, it can be concluded that the layered structured particulates being more spherical (roundness = 0.83), flowed better at 23.69 g/s and achieved a slightly lower Hausner Ratio of 1.045 compared to the matrix structured particulates. The latter, while being considerably less spherical (roundness = 0.71) still flowed exceptionally well (flow rate of 20.45 g/s) and their compressibility was at almost the same rate as the layered pellets’ (Hausner Ratio of 1.047). Both types of investigated samples were larger than a typical pharmaceutical pellet with Feret diameters of about 3.6 and 3.5 mm, while their projected area was about 8.0 and 6.6 mm^2^, respectively. However, these dimensions were deemed acceptable to load the particulates into plastic drinking straws, and for the particulates to remain inside those straws while the dissolution tests were carried out. Both types disintegrated in less than 3 min. The disintegration of the particles within 3 min was important because this time interval is also determined by the European Pharmacopoeia as the maximum acceptable disintegration time of orodisperse tablets [29].

**Figure 3:**
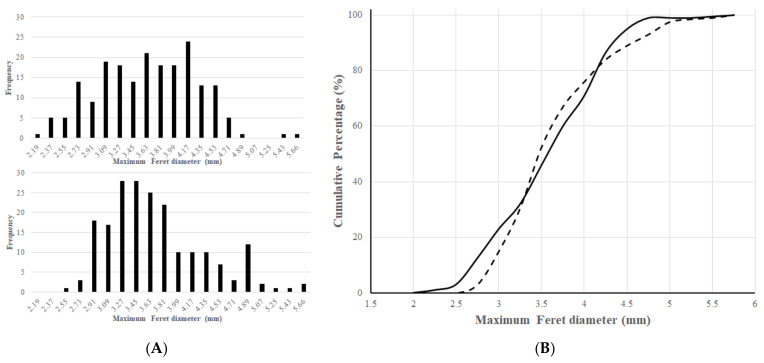
Particle size distribution of the matrix ((**A**) and continuous line) and layered ((**B**) and dashed line) particles.

The PSD of the two different particles were calculated and presented in Figure 3. The results show that both particles have a unimodal distribution. The distribution of the matrix system is between the range of 2–4 mm (Figure 3A), while the layered particles give a sharper peak at 3 mm (Figure 3B). From this, it also can be seen that our particles were larger than a conventional pellet, but this property only facilitated their suitability for being loaded into and for being retained in the straws.

**Figure 4:**
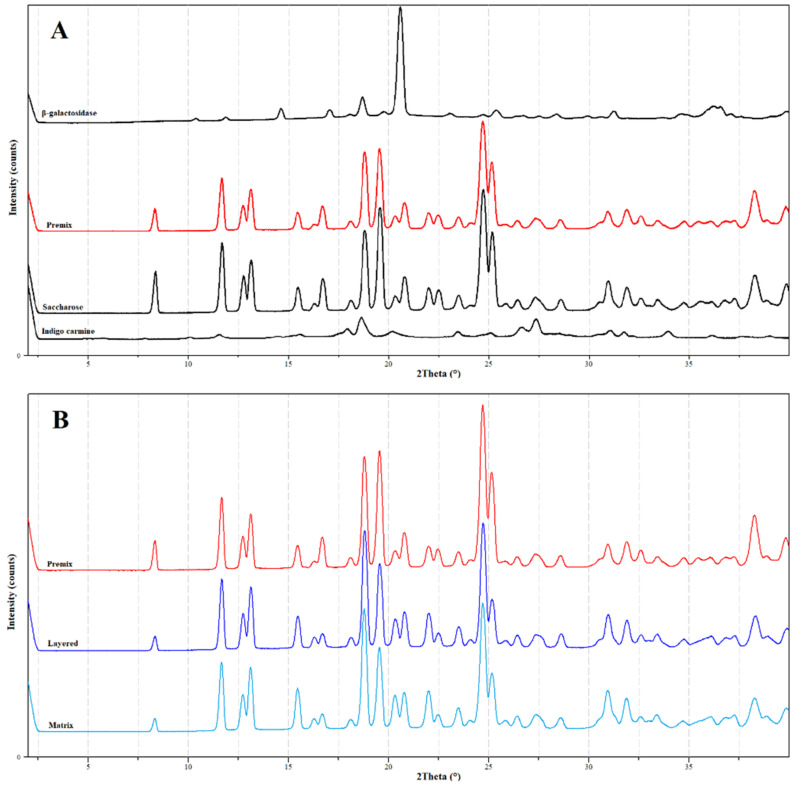
XRD profiles of the used materials (**A**) (β-galactosidase, saccharose and indigo carmine in black), (**B**) premixed powder (red), powdered layered particles (blue) and powdered matrix particles (light blue).

As the result of the X-ray crystallographic examinations, it has been established that all the used raw materials are crystalline (Figure 4A). After the formulation the theta values were retained (Figure 4B), this shows that the materials remained in crystalline form, although, the signals from the used sugar (saccharose) in the prepared pellets and in the premix completely suppress the signals from the rest of the ingredients due to the large difference in ratio (less than 1% *w*/*w* β-galactosidase and indigo carmine in the formula).

### 3.2. Characterization of Straws

**Figure 5:**
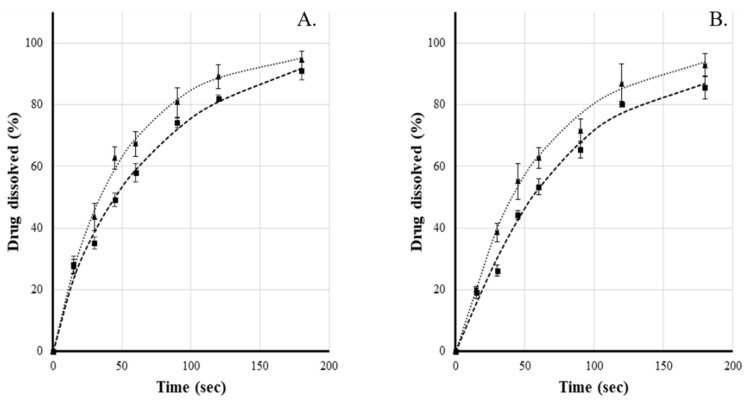
Indigo carmine (**A**) and β-galactosidase (**B**) release profile of straw containing matrix (■) and layered pellets (▲). T = 20 °C; 40 Hz.

Since no large discrepancy can be observed between the two different types of particulates (matrix, layered) in the disintegration study, for this reason, as expected, the dissolution profile of lactase and indigo carmine had a similar shape in case of both heterogeneous and homogeneous particles (Figure 5). The straws were loaded by hand with a filling weight of average 5.0383 g (±0.0697, *n* = 20). The activity of the active ingredient was then measured back from 1-1 g load relative to the nominal weight. For the two different types of filling (matrix and layered), it was 98.22% and 98.59%, respectively (±1.70 and 0.86, *n* = 10-10). These values are in accordance with the requirements of the 10th European Pharmacopoeia for single-dose preparations [25]. The flow resistance of the different charges was also similar, however, there was no observable increase compared to the unfilled straw. For this reason, the volume of the flow did not change in case of the loaded straws. The difference in mass compared to the unloaded straw is given by the rapidly dissolving substance during the measurement, the increase in mass correlates well with the values obtained during the dissolution studies (Table 4).

**Figure 6:**
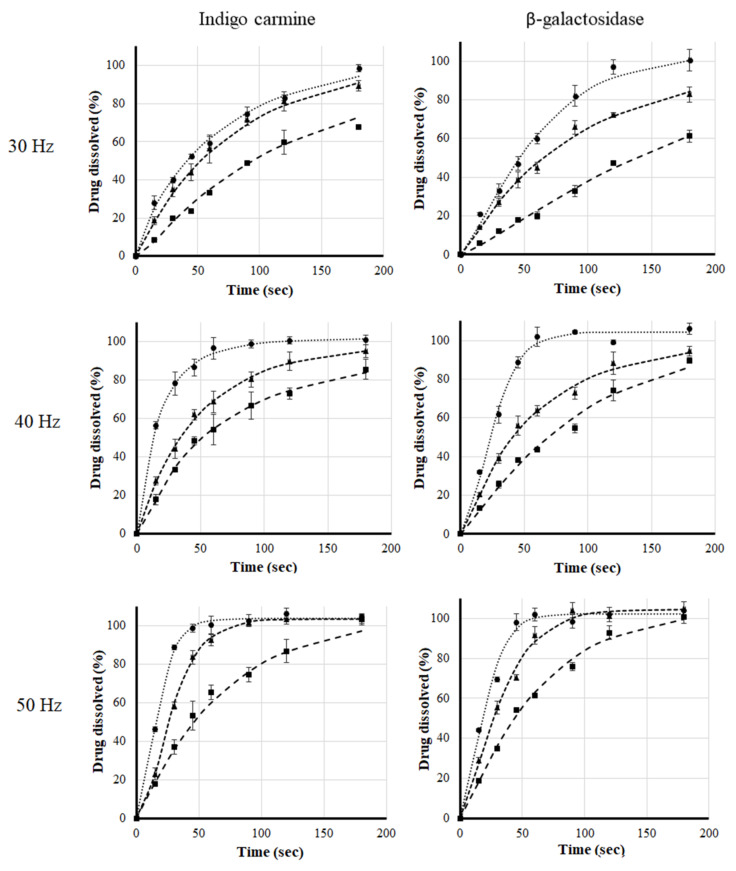
Indigo carmine (1st column) and β-galactosidase (2nd column) release profile (●; ■; ▲; *n* = 3 ± SD) with fitted Weibull function (dotted lines). The pump frequencies are 30 Hz; 40 Hz or 50 Hz, and the fluid exhibiting different temperatures: 5 °C (■); 20 °C (▲) and 35 °C (●) during the simulation.

Straws filled with the layered-structured particles were used to investigate the effect of liquid temperature and flow rate on the dissolution profiles in the simulation. The results of using a liquid of different temperatures and “sipping” at various frequencies are shown in Figure 6. It can be seen that for both β-galactosidase and indigo carmine, the applied frequency and the temperature of the liquid had a significant effect on the release profile.

Table 5 shows the kinetic parameters (*t_0_*, *τ_d_*, *b*) determined using the Weibull distribution function. Considering the dissolution rate, the data indicate that *τ_d_* is strongly dependent on fluid temperature and on the pump frequency. The polynomial function was used to model the effect of independent variables on *τ_d_*. During the indigo carmine release of trial No. 4, where the value of *b* < 0.69 the release follows the law of Fickian diffusion in fractal space. The value of b between 0.75 and 1 indicate diffusion in normal Euclidian substrate with the contribution of another release mechanism. *b* = 1 means that the first order release obeys Fick’s first law of diffusion and if *b* > 1, a complex mechanism causes the sigmoid dissolution curve [30].

**Figure 7:**
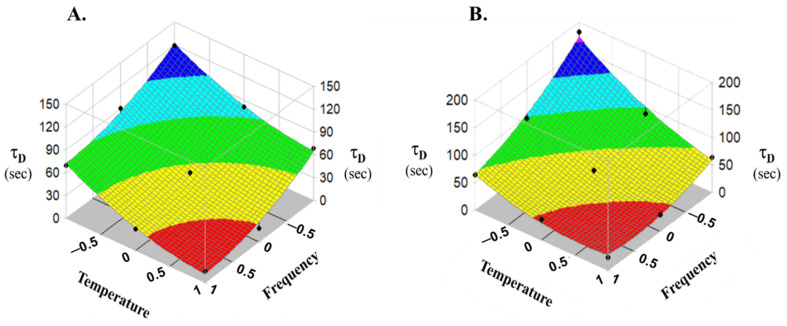
Surface plot of the effects of frequency and temperature on the release of indigo carmine (**A**) or β-galactosidase (**B**).

Figure 7 shows the surface fitted to nine points by the polynomial equation (Equation (2)). The results of the multiple regression analysis with statistical evaluation for significance and probabilities at 95% confidence interval are shown in Table 6. The values of the coefficients of *x*_1_, *x*_2_ relate to the effects of pump frequency and temperature of the medium on the *τ_d_* value.

For both indigo carmine and lactase, the sign of the main effect of the two independent variables is negative. Meaning that, increasing both the temperature of the liquid and the value of the pump frequency decreases the value of *τ_d_*, thus, it can be characterized by a faster release profile, but the effects are independent of each other. The magnitude of the values of these coefficients (*b*_1_ and *b*_2_) is similar. The combined effect of the two independent variables is only valid for lactase, as the interaction parameters indicate (*b*_12_). The resultant equations (Equations (3) and (4)) represent the significant effect independent variables have on the *τ_d_* time parameter.

(3) $$\tau_{indigo\;carmine\;}=44.63-26.055x_{1}-29.705x_{2}\;\left(R=0.9919\right)$$

(4) $$\tau_{\beta -galactosidase\;}=50.34-34.817x_{1}-37.052x_{2}+19.36x_{1}x_{2}\;\left(R=0.9880\right)$$

## 4. Conclusions

Nowadays, the development of age-appropriate oral dosage forms has come to the forefront. For younger children, liquid dosage forms are preferred. For this reason, the present study examined an enzyme-containing straw. Rapidly disintegrating particulates containing lactase enzyme that can be loaded in drinking straws has been successfully prepared by applying two different technological methods. The application of a child-favoured medical formula was simulated, and in the statistical analysis it was found that both the temperature of the fluid used and the speed of the fluid intake throughout admission have almost the same and significant effect on drug release rate.

## Figures and Tables

**Table 1:** The results of the quality control measurements.

	Indigo Carmine		β-Galactosidase	
	QC1 (1 µg/mL)	QC2 (10 µg/mL)	QC1 (0.25 µg/mL)	QC2 (1 µg/mL)
Average	0.9762	10.0981	0.2571	0.9748
Accuracy	97.62	100.98137	102.81	97.48
Precision	9.463	4.028	3.588	0.7250

**Table 3:** Results (mean ± SD) of image analysis, flowability and compressibility measurements of pellet formulations. R = Roundness (*n* = 200), F = Maximum Feret diameter (*n* = 200), A = Projected Area (*n* = 200), FR = Flow Rate (*n* = 3), BD = Bulk Density, TD = Tapped Density, HR = Hausner ratio, LDT = Longest Disintegration Time.

	R	F (mm)	A (mm^2^)	Flowability (g/s)	BD (cm^3^)	TD (cm^3^)	HR	LDT (s)
Layered pellets	0.83 ± 0.102	3.61 ± 0.622	7.976 ± 2.191	23.69 ± 0.23	117	112	1.045	135
Matrix pellets	0.71 ± 0.134	3.56 ± 0.634	6.639 ± 1.735	20.45 ± 0.38	134	128	1.047	158

**Table 4:** The flow rate generated by the different frequencies for various samples (mean ± SD, *n* = 3).

Flow Rate (g/min)		Frequency (Hz)		
		30	40	50
	Unloaded	72.06 ± 0.62	99.7 ± 1.39	140.76 ± 0.70
Types of partciles	Layered	75.23 ± 1.37	103.4 ± 0.48	144.6 ± 0.55
	Matrix	74.00 ± 1.15	103.03 ± 0.39	144.16 ± 0.70

**Table 5:** Symbolised levels of independent variables of the experimental design and kinetic parameters of dissolution estimated according to the Weibull distribution function (Mean ± SD).

Substance	Trial No.	Coded Value of *x*_1_	Coded Value of *x*_2_	*t*_0_ (s)	*τ_d_* (s)	*b*	R
Indigo carmine	1	+1	−1	1.08 ± 1.52	69.57 ± 8.39	1.02 ± 0.01	0.997
	2	+1	0	5.07 ± 3.00	28.21 ± 3.59	1.31 ± 0.08	0.999
	3	+1	+1	5.28 ± 0.50	15.00 ± 0.00	1.19 ± 0.14	0.998
	4	0	−1	9.10 ± 1.19	90.35 ± 7.77	0.65 ± 0.00	0.998
	5	0	0	1.07 ± 1.85	48.29 ± 4.06	0.92 ± 0.07	0.999
	6	0	+1	1.94 ± 3.36	17.69 ± 2.35	0.80 ± 0.21	0.996
	7	−1	−1	5.17 ± 0.13	119.82 ± 1.97	0.98 ± 0.01	0.988
	8	−1	0	2.42 ± 4.2	80.48 ± 5.61	0.91 ± 0.21	0.996
	9	−1	+1	0.00 ± 0.00	68.80 ± 1.59	0.86 ± 0.07	0.996
β-galactosidase	1	+1	−1	1.91 ± 1.88	63.43 ± 1.25	1.06 ± 0.09	0.997
	2	+1	0	0.00 ± 0.00	37.42 ± 1.39	1.32 ± 0.07	0.996
	3	+1	+1	0.00 ± 0.00	23.67 ± 0.51	1.47 ± 0.07	0.992
	4	0	−1	0.00 ± 0.00	95.78 ± 1.46	1.09 ± 0.05	0.993
	5	0	0	5.09 ± 3.72	56.80 ± 7.70	0.85 ± 0.14	0.997
	6	0	+1	0.00 ± 0.00	30.42 ± 2.12	1.59 ± 0.19	0.996
	7	−1	−1	0.00 ± 0.00	181.58 ± 7.26	1.20 ± 0.12	0.996
	8	−1	0	6.64 ± 0.73	87.46 ± 4.23	0.88 ± 0.024	0.999
	9	−1	+1	0.00 ± 0.00	64.40 ± 3.84	1.24 ± 0.12	0.991
x_1_: rotation frequency of the pump; x_2_: temperature							

**Table 6:** Statistical analysis of models.

Substance	Model F-Value	Parameter	Coefficients					
			*b*_0_	*b*_1_	*b*_2_	*b*_11_	*b*_22_	*b*_12_
Indigo carmine	36.716 (*p* > 0.0068)	Value	44.63	−26.055	−29.705	11.54 *	11.21 *	−0.89 *
		Std Error	5.470	2.996	2.996	5.190	5.190	3.670
		*p* > ItI	0.004	0.003	0.002	0.113	0.120	0.824
β-galacto-sidase	24.564 (*p* > 0.0122)	Value	50.34	−34.817	−37.052	15.323 *	15.99 *	19.36
		Std Error	9.021	4.941	4.941	8.558	8.588	6.051
		*p* > ItI	0.011	0.006	0.005	0.171	0.159	0.049

*: not significant.

## Data Availability

Not applicable.
